# Incidence of heart valve disease in women treated with the ergot-derived dopamine agonist bromocriptine

**DOI:** 10.1186/s12872-021-02439-y

**Published:** 2021-12-28

**Authors:** Marianne F. Clausen, Rasmus Rørth, Christian Torp-Pedersen, Lucas Malta Westergaard, Peter E. Weeke, Gunnar Gislason, Lars Køber, Emil Fosbøl, Søren Lund Kristensen

**Affiliations:** 1 Department of Cardiology, Rigshospitalet, University of Copenhagen, Blegdamsvej 9, 2100 Copenhagen, Denmark; 2 Department of Clinical Investigation and Cardiology, Nordsjaellands Hospital, Hilleroed, Denmark; 3 Department of Cardiology, Gentofte/Herlev University Hospital, Copenhagen, Denmark; 4 Department of Clinical Medicine, Faculty of Health and Medical Sciences, University of Copenhagen, Copenhagen, Denmark

**Keywords:** Bromocriptine, Ergot-derived dopamine agonist, Heart valve disease, Hyperprolactinemia

## Abstract

**Background:**

Ergot-derived dopamine agonists are thought to induce fibrotic changes in cardiac valve leaflets. We sought to determine the incidence of heart valve disease in women treated with bromocriptine compared with age and sex matched controls from the background population.

**Methods:**

In nationwide Danish registries we identified female patients treated with bromocriptine in the period 1995–2018. Patients were included at date of second redeemed prescription and were matched 1:5 with controls from the background population based on age, sex and year of inclusion by use of incidence density sampling. The outcomes were hospital admission for or outpatient diagnosis of heart valve disease, and death as competing risk. Incidence rates, cumulative incidence curves, and adjusted cox-proportional hazard models adjusted for cardiovascular risk factors were used to assess outcomes in bromocriptine users versus controls.

**Results:**

A total of 3035 female bromocriptine users and 15,175 matched controls were included. Median age at inclusion was 32 years (Q1–Q3, 28–37 years). Both bromocriptine users and controls had few comorbidities and low use of concomitant pharmacotherapy. Within 10 years of follow-up, 11 patients (0.34%, 95% CI 0.13–0.55%) and 44 controls (0.29%, 95% CI 0.20–0.37) met the primary endpoint of heart valve disease, *p* = 0.63. The adjusted cox regression analysis yielded a hazard ratio of 0.96 (95% confidence interval (CI) 0.55–1.69, *p* = 0.89).

**Conclusions:**

Treatment initiation with ergot-derived dopamine agonist bromocriptine in younger women with few comorbidities, was associated with a low absolute long-term risk of heart valve disease, not significantly different from the risk in age and sex matched population controls. Thus, indicating a low clinical yield of pre-treatment echocardiographic screening in this patient population in accordance with current guidelines.

**Supplementary Information:**

The online version contains supplementary material available at 10.1186/s12872-021-02439-y.

## Key points

- The ergot-derived dopamine agonist cabergoline are thought to induce fibrotic changes in valve leaflets.
- Bromocriptine is perceived as a safe cardiovascular alternative to cabergoline, but the knowledge on which this is based is sparse.
- In our study the use of bromocriptine was not associated with an increased risk of heart valve disease relative to age and sex matched population controls.
- 10-year incidence of heart valve disease was low and did not differ between bromocriptine treated patients and controls; 0.34% (95% CI 0.13–0.55) vs 0.29% (95% CI 0.20–0.37), respectively *p* = 0.63.

## Background

Ergot-derived dopamine agonists are thought to induce fibrotic changes in valve leaflets, causing thickening and stiffening of valves and thereby valve regurgitation [1]. This pathophysiological link to an increased heart valve disease risk may represent a class effect, related to stimulation of serotonin receptors, including the 5-hydroxy-tryptamine 2B receptors (5-HT2B), which also are found in cardiac tissue, and subclinical heart valve disease has been described in patients treated with bromocriptine [2–5]. Other ergot-derived dopamine agonists, such as cabergoline and pergolide, exert full agonistic activity on the 5-HT2B receptors whereas bromocriptine only shows partial agonistic activity [2, 6].

Ergot-derived dopamine agonists are in general considered safe for long-term treatment of hyperprolactinemia due to the lower doses used compared with the dosages given to patients with Parkinson’s disease [7–11]. However, echocardiographic screening for heart valve disease are currently recommended for patients with hyperprolactinemia at treatment initiation and periodically during cabergoline therapy due to a suggested increased prevalence of mild to moderate regurgitation in the tricuspid, aortic and mitral valves in these patients[12, 13]. Previously, smaller studies have showed no signal of an association between bromocriptine and an increased incidence of heart valve disease, and bromocriptine is therefore perceived as a safe cardiovascular alternative to cabergoline with no need for echocardiographic screening [8, 12–14].

As bromocriptine is used frequently in an otherwise young and healthy, primarily female population, any correlation with an increased risk of heart valve disease is clinically important. We therefore examined the relation between bromocriptine use and heart valve disease in a nationwide Danish cohort with median follow-up of around 20 years.

## Methods

### Data sources

Danish nationwide administrative registries were used to collect data at individual levels by use of a unique personal identification number which is assigned to all residents in Denmark. For this study, three nationwide registers were linked on an individual level to obtain information on all Danish residents aged 18 years or older and who had claimed prescriptions of bromocriptine between 1 July 1995 to 26th June 2018. The Danish National Patient Register holds information about all admissions to Danish hospitals since 1978, and outpatients visits since 1990, including diagnoses coded according to the International Classification of Diseases, eighth edition (ICD-8) and ICD-10 and all procedures, surgeries included, are coded according to the Nordic Medico-Statistical Committee (NOMESCO) classification [15]. The Danish National Prescription Registry, which holds information on all claimed prescriptions in Denmark since 1995, ensuring complete data on date of dispensing, strength of the tablets, number of pills dispensed and of cause the prescribed drug grouped according to the Anatomical Therapeutic Chemical (ATC) codes [16]. And the civil register contains information about vital status of all Danish residents [17]. Register-based studies in which individuals cannot be identified do not require ethical approval in Denmark.

### Study population

We included female patients aged 18 years or older treated with bromocriptine and matched controls identified from the background population. Men treated with bromocriptine were excluded due to different indications for treatment, older age at treatment initiation, and differences in clinical characteristics [18]. Patients entered the study cohort when claiming a second prescription of bromocriptine with this date defining the study inclusion date. Each patient was matched by age and sex to 5 matched controls, and patients and controls were followed from index date until occurrence of the first of following events: hospitalization for heart valve disease or an outpatient contact with a heart valve diagnosis, death, or end of study period (31th June 2018). We excluded patients who prior to study entry, had a history of: heart valve disease, rheumatic heart disease, rheumatic fever, chronic heart failure, congenital heart and valve disease, endocarditis, carcinoid syndrome, Parkinson’s disease, pergolide-, levodopa-, cabergoline-, quinagolide-use, or patients who had been treated with medications that may induce fibrosis (fenfluramine, dexfenfluramine, ergotamine) [19]. To ensure patients were naive bromocriptine users, we excluded patients who claimed a prescription in the first half of 1995 (first year of full data coverage in the registry). All patients were individually risk set matched by age, sex and year of inclusion with 5 controls from the background population. In a sensitivity analysis we restricted the population to patients who claimed five or more prescriptions with the date of the fifth prescription claim set as index date. We also repeated the analysis with a more detailed matching of each patient by age, sex, prior diabetes, deep venous thrombosis, bleeding, chronic renal failure, ischemic heart disease, stroke, chronic obstructive lung disease and ongoing use of beta blockers, loop diuretics and aspirin, with two controls from the background population.

The majority of patients (82%) had no prior in or outpatient hospital diagnosis of hyperprolactinaemia before initiating bromocriptine treatment. We had no access to patient charts from the general practitioner but we assume these patients were treated by their general practitioner as we included other in and out hospital diagnoses which could indicate bromocriptine treatment.

Comorbidities and concomitant pharmacotherapy were identified by assessing all hospital discharge codes prior to index and information on claimed prescriptions one year before index date. ICD, procedure, and ATC codes used in this study are listed in the Additional file 1: Tables 2 and 3.

### Outcomes

The primary outcome was development of heart valve disease as defined by an outpatient clinic visit or hospital admission with a diagnosis of heart valve disease or valvular heart surgery. As secondary outcomes we assessed the risk of valvular heart surgery as proxy for the severity of heart valve disease.

### Statistical analysis

Baseline characteristics for bromocriptine-treated patients and the control cohort were described by use of numbers and percentages for categorical variables and medians and interquartile ranges for continuous variables. Differences between the bromocriptine group and controls were obtained by use of Chi-square test for categorical variables.

Cumulative incidence curves for heart valve disease, with death as a competing risk, were estimated, and differences between patients and control cohort were compared using Gray’s test. Crude incidence rates were calculated. Cause-specific Cox regression were used to compare risk of heart valve disease between patients and the control cohort. Cox regression analyses were adjusted for age at index, year of inclusion and history of hypertension, ischemic heart disease, acute myocardial infarction and diabetes mellitus. The variables adjusted for were chosen based on clinical relevance and known prognostic importance in heart valve disease. The variable age and comorbidities (hypertension, ischemic heart disease, acute myocardial infarction and diabetes) were tested for interactions with the use of bromocriptine in relation to both outcomes and, unless stated otherwise, found absent. Interactions were considered significant if they yielded a *p*-value < 0.05. Log (-log(survival)) curves were used to evaluate the proportional hazard assumption. The assumption of linearity of age was tested by including a variable of age squared.

Furthermore, the interaction between patients with and without an ICD-code for hyperprolactinemia in addition to use of bromocriptine were tested for all outcomes.

In a supplementary analysis, we calculated the accumulated dosage of claimed bromocriptine for patients with and without a heart valve disease diagnosis. Differences between the groups were obtained by use of Wilcoxons test.

Results were considered significant if *p* < 0.05. The SAS statistical software package, version 9.4 (SAS Institute, Cary, North Carolina; USA) and R, version 3.5.0 (R development Core Team) were used for all analyses.

## Results

### Baseline characteristics

**Fig. 1:**
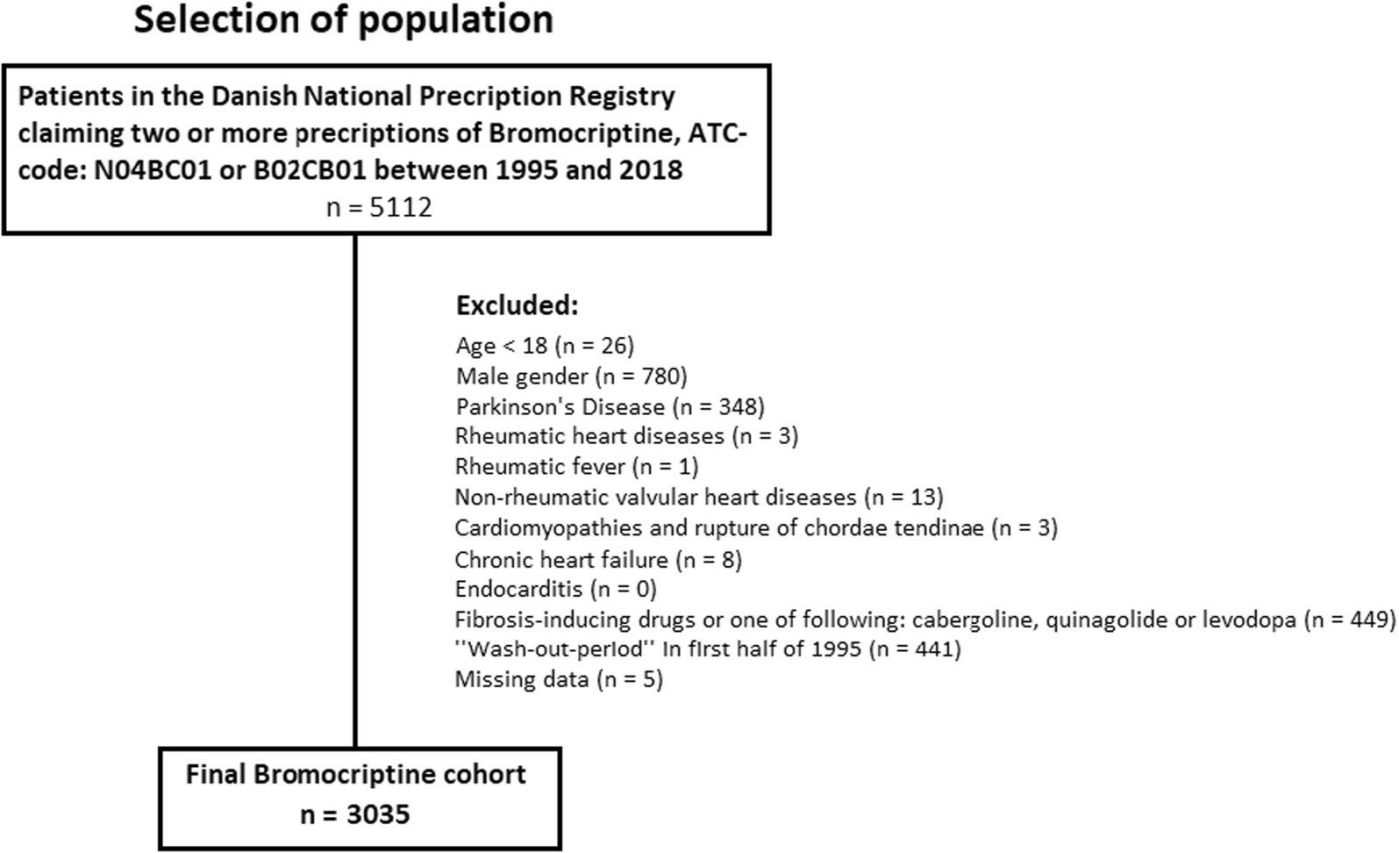
Flow chart displaying patient selection

A total of 3035 female patients treated with bromocriptine between 1995 and 2018 were included in the study (Fig. 1) and matched with 15,175 controls. Clinical characteristics of patients and the matched control cohort are summarized in Table 1. Median age at index was 32 years (Q1-Q3, 28–37 years) and both groups had few comorbidities and low use of pharmacotherapy. The bromocriptine treated patients were more likely to have prior hypertension (75 (2.5%) vs 220 (1.4%)), diabetes (42 (1.4%) vs 101 (0.7%)) and chronic obstructive lung disease (21 (0.7%) vs 42 (0.3%)) with *p*-values < 0.0001, and patients were more frequent users of beta-blockers (82 (2.7%) vs 247 (1.6%), *p* < 0.0001) and thiazides (84 (2.8%) vs 270 (1.8%), *p* = 0.0004). In the sensitivity analysis with additional matching for major comorbidities and pharmacotherapy use, we included 2903 female patients and 5729 matched controls (Additional file 1: Table 1).

**Table 1:** Baseline characteristics of bromocriptine treated patients and matched controls †

Variable	Bromocriptine (n = 3035)	Controls (n = 15,175)	*p*-value
Age at onset (year)	31.7 [27.8, 36.8]	31.7 [27.7, 36.8]	-
**Comorbidities, n (%)**			
Ischemic heart disease	20 (0.7)	64 (0.4)	0.11
Acute myocardial infarction	5 (0.2)	20 (0.1)	0.86
Atrial fibrillation	8 (0.3)	27 (0.2)	0.45
Ischemic stroke	15 (0.5)	50 (0.3)	0.22
Transient ischemic attack	7 (0.2)	19 (0.1)	0.25
Embolism	0 (0.0)	9 (0.1)	0.37
Pulmonary embolism	4 (0.1)	15 (0.1)	0.84
Deep vein thrombosis	16 (0.5)	39 (0.3)	0.02
Atherosclerosis	6 (0.2)	15 (0.1)	0.24
Coagulopathy	5 (0.2)	26 (0.2)	1.0
Hypertension	75 (2.5)	220 (1.4)	0.0001
Diabetes	42 (1.4)	101 (0.7)	0.0001
Diagnosis of alcohol misuse	53 (1.7)	123 (0.8)	0.0001
Chronic obstructive lung disease	21 (0.7)	42 (0.3)	0.0007
Malignancy	37 (1.2)	178 (1.2)	0.90
Chronic renal disease	16 (0.5)	52 (0.3)	0.17
Abnormal liver function	14 (0.5)	58 (0.4)	0.64
**Medication, n (%)**			
Beta-blockers	82 (2.7)	247 (1.6)	0.0001
Calcium channel blockers	38 (1.3)	132 (0.9)	0.06
RAS inhibitors	38 (1.3)	164 (1.1)	0.47
Vasodilators	0(0.0)	0 (0.0)	1.0
Antiadrenergic drugs	8 (0.3)	8 (0.1)	0.001
Thiazides	84 (2.8)	270 (1.8)	0.0004
Loop diuretics	57 (1.9)	117 (0.8)	0.0001
Spironolactone	11 (0.4)	14 (0.1)	0.0007
Diuretics, combination	12 (0.4)	49 (0.3)	0.65
Digoxin	6 (0.2)	27 (0.2)	1.0
Statins	18 (0.6)	74 (0.5)	0.54
Antidiabetics	55 (1.8)	107 (0.7)	0.0001
Aspirin	33 (1.1)	108 (0.7)	0.04
ADP inhibitors	0 (0.0)	0 (0.0)	1.0
Anticoagulants	5 (0.2)	23 (0.2)	1.0

^†^patients and controls were matched for age and sex

*p*-values were obtained by chi-square test for comparison between patients and controls

*IQR* Interquartile range

Pharmaceuticals: Claimed prescription within 366 days before baseline date

*RAS* Renin-Angiotensin system

### Heart valve disease

The median follow-up for patients were 18.2 years (Q1-Q3, 14.7–20.7 years) vs 18.2 years (Q1-Q3, 14.8–20.8 years) for controls. During follow-up 16 of 3035 (0.56%) patients and 91 of 15,175 (0.60%) controls were diagnosed with heart valve disease. The incidence rate of heart valve disease among patients treated with bromocriptine was 0.31 per 1000 patient years (PY) (95% CI 0.20–0.51) compared with 0.35 per 1000 PY in the control cohort (95% CI 0.28–0.43).

**Fig. 2:**
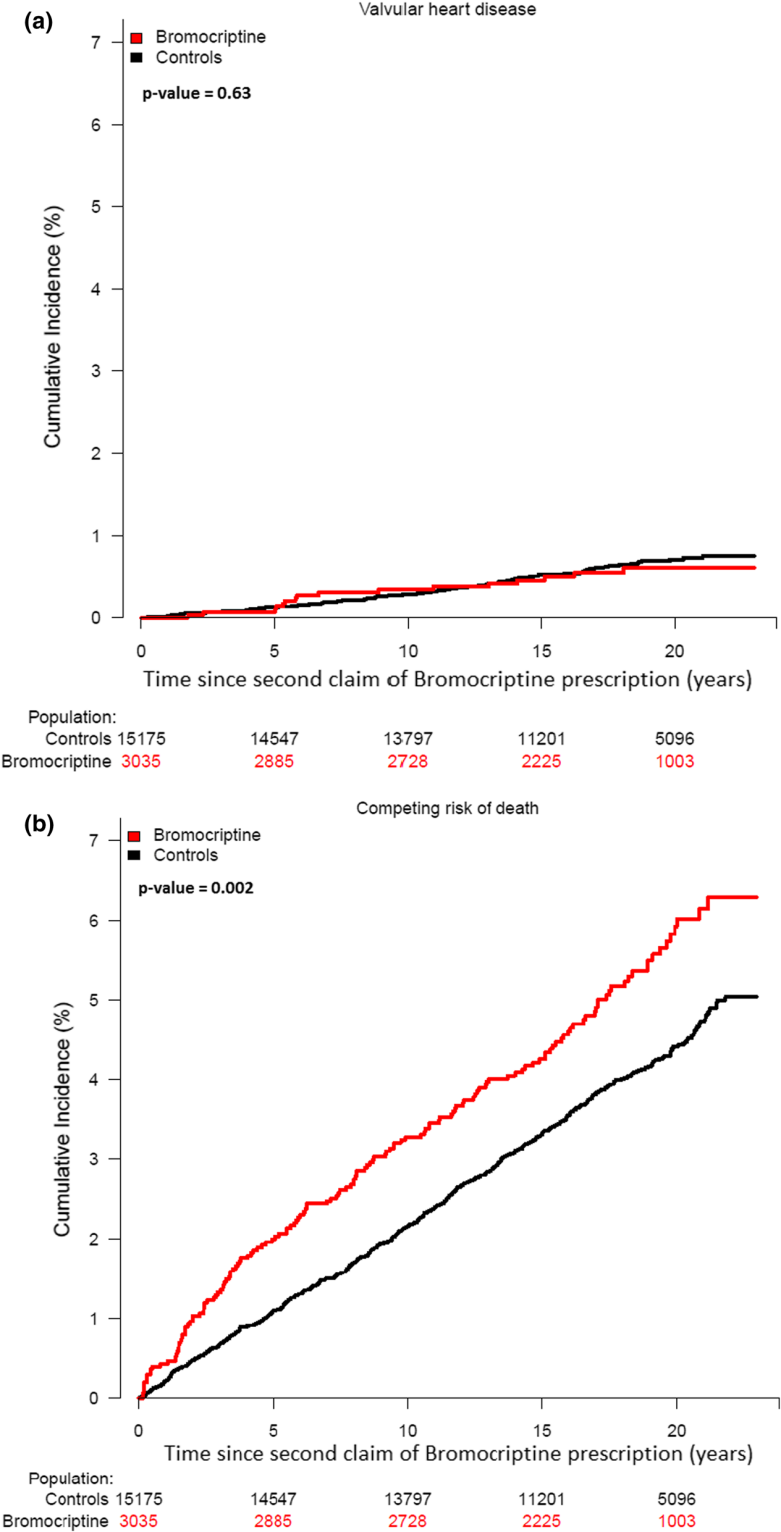
**a** Cumulative incidence of valvular heart disease with death as a competing risk among bromocriptine-treated patients and matched controls. **b** Cumulative incidence of competing risk of death among bromocriptine-treated patients and matched controls

The cumulative incidence of heart valve disease with the competing risk of death are shown in Fig. 2a, b. The 10-year incidence of heart valve disease was 0.34% (95% CI 0.13–0.55) for patients vs 0.29% (95% CI 0.20–0.37) for controls, while the 20-year incidence of heart valve disease was 0.61% (95% CI 0.31–0.91) for patients and 0.70% (95% CI 0.55–0.85) for the control cohort (*p* = 0.63). Competing risk of death was 6.0% (95% CI 5.0–7.0) among patients treated with bromocriptine and 4.4% (95% CI 4.0–4.8) for the control cohort, *p* = 0.002.

The unadjusted cox regression model for the risk of heart valve disease in patients vs. controls yielded a hazard ratio (HR) of 1.06 (95% CI 0.61–1.84, *p* = 0.85), and the adjusted analysis yielded a HR of 0.96 (95% CI 0.55–1.69, *p* = 0.89).

In the sensitivity analysis which included matching on additional baseline variables, we found incidence rates of heart valve disease of 0.26 per 1000 PY (95% CI 0.15–0.44) compared with 0.27 per 1000 PY in the control cohort (95% CI 0.19–0.40), and this analysis yielded similar although slightly lower estimates for 10- and 20-year incidence of heart valve disease. The competing risk of death, in this analysis was 5.4% (95% 4.5–6.4) among patients treated with bromocriptine and 4.2% (95% 3.6%-4.8%) for the controls, *p* = 0.02.

### Heart valve surgery

**Fig. 3:**
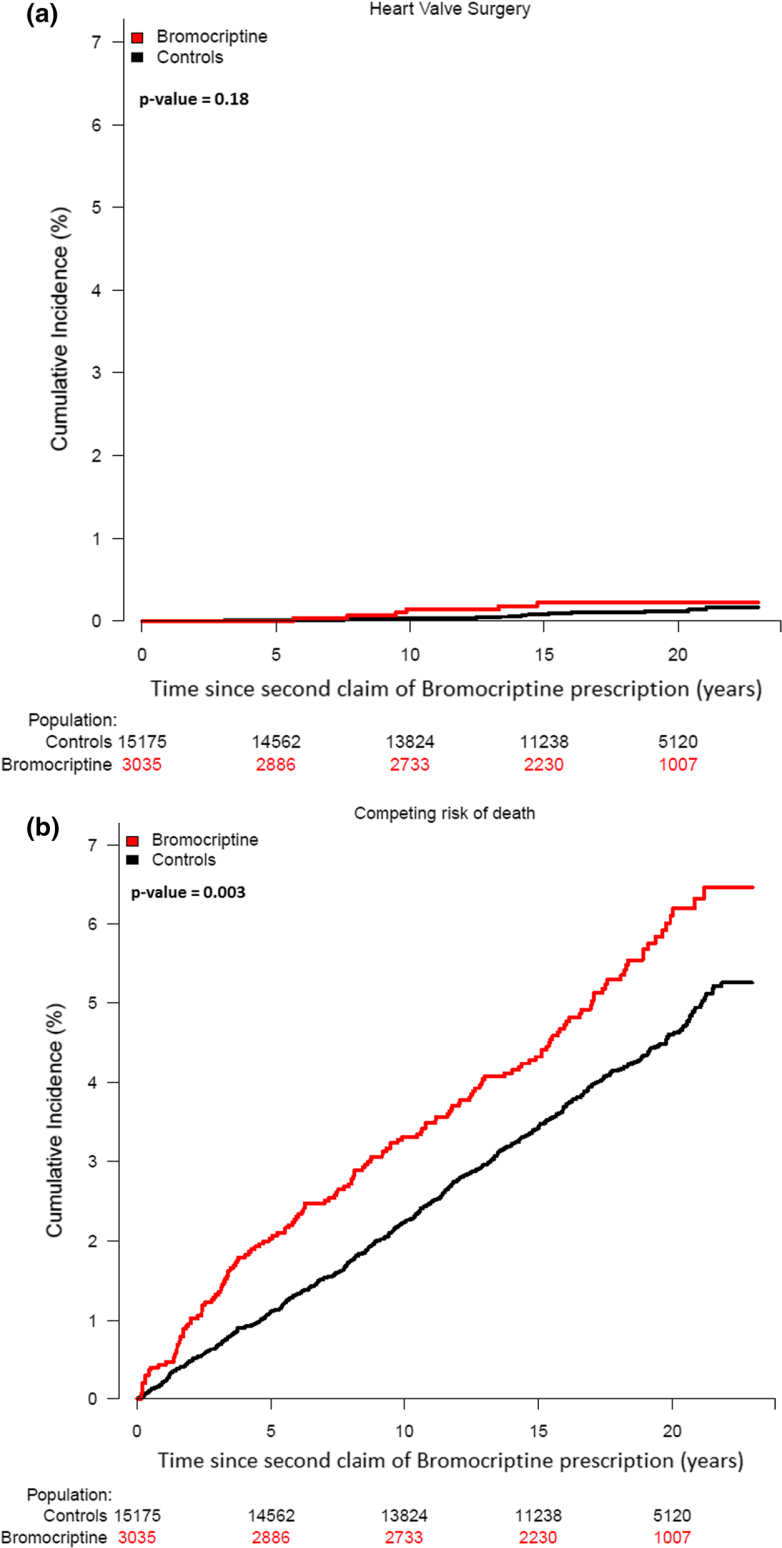
**a** Cumulative incidence of heart valve surgery among bromocriptine-treated patients and matched controls. **b** Cumulative incidence of competing risk of death among bromocriptine-patients and matched controls

During the study period, 6 patients (0.20%) and 16 controls (0.11%) underwent heart valve surgery. The 20-year incidence of heart valve surgery was 0.22% (95% CI 0.04–0.40) for patients and 0.11% (95% CI 0.05–0.18) for the control cohort; *p* = 0.18 (Fig. 3a). Competing risk of death was 6.2% (95% CI 5.2–7.2) among patients and 4.6% (95% CI 4.2–5.0) among controls; *p* = 0.003 (Fig. 3b).

Unadjusted cox regression model for heart valve surgery yielded a HR of 2.05 (95% CI 0.78–5.43, *p* = 0.15), while adjusted analysis yielded a HR of 1.88 (95% CI 0.68–5.19, *p* = 0.22), for those who initiated bromocriptine treatment vs matched controls.

### Diagnosis of hyperprolactinaemic disorders

Of 3035 patients who initiated bromocriptine treatment, 544 (18%) had a prior hospital discharge diagnosis of hyperprolactinaemia. We found no significant difference in the risk of heart valve disease between patients with and without a prior diagnosis (*p* = 0.51).

### Long-term use of bromocriptine

In a sensitivity analysis, we identified 603 patients who claimed five or more bromocriptine prescriptions during follow-up. In this group, 5 patients (0.83%) were diagnosed with heart valve disease, while 1 (0.17%) patient underwent heart valve surgery. These patients were characterized by more comorbidity and use of pharmacotherapy at baseline (Table 2).

**Table 2:** Baseline characteristics of patients claiming > 5 and < 5 bromocriptine prescriptions

Variable	> 5 claimed bromocriptine percriptions (n = 603)	< 5 claimed bromocriptine percriptions(n = 2432)	*p*-value
Age at onset (year)	35.0 [28.7, 44.5]	31.3 [27.6, 35.5]	< 0.0001
**Comorbidities, n (%)**			
Ischemic heart disease	7 (1.2)	13 (0.5)	0.16
Acute myocardial infarction	≤ 3 (NA)	≤ 3 (NA)	NA
Atrial fibrillation	5 (0.8)	≤ 3 (NA)	NA
Ischemic stroke	7 (1.2)	8 (0.3)	0.02
Transient ischemic attack	4 (0.7)	≤ 3 (NA)	NA
Embolism	0 (0.0)	0 (0.0)	NA
Pulmonary embolism	0 (0.0)	4 (0.2)	0.71
Deep vein thrombosis	≤ 3 (NA)	13 (0.5)	NA
Atherosclerosis	6 (1.0)	0 (0.0)	< 0.0001
Coagulopathy	≤ 3 (NA)	4 (0.2)	NA
Hypertension	41 (6.8)	34 (1.4)	< 0.0001
Diabetes	18 (3.0)	24 (1.0)	0.0004
Diagnosis of alcohol misuse	15 (2.5)	38 (1.6)	0.17
Chronic obstructive lung disease	8 (1.3)	13 (0.5)	0.07
Malignancy	18 (3.0)	19 (0.8)	< 0.0001
Chronic renal disease	5 (0.8)	11 (0.5)	0.41
Abnormal liver function	6 (1.0)	8 (0.3)	0.07
**Medication, n (%)**			
Beta-blockers	34 (5.6)	48 (2.0)	< 0.0001
Calcium channel blockers	19 (3.2)	19 (0.8)	< 0.0001
RAS inhibitors	20 (3.3)	18 (0.7)	< 0.0001
Vasodilators	0 (0.0)	0 (0.0)	NA
Antiadrenergic drugs	≤ 3 (NA)	6 (0.2)	NA
Thiazides	45 (7.5)	39 (1.6)	< 0.0001
Loop diuretics	26 (4.3)	31 (1.3)	< 0.0001
Spironolactone	7 (1.2)	4 (0.2)	0.001
Diuretics, combination	7 (1.2)	5 (0.2)	0.003
Digoxin	0 (0.0)	6 (0.2)	0.48
Statins	12 (2.0)	6 (0.2)	< 0.0001
Antidiabetics	22 (3.6)	33 (1.4)	0.0003
Aspirin	18 (3.0)	15 (0.6)	< 0.0001
ADP inhibitors	0 (0.0)	0 (0.0)	NA
Anticoagulants	≤ 3 (NA)	≤ 3 (NA)	NA

*p*-values were obtained by chi-square test for comparison

*IQR* Interquartile range

Pharmaceuticals: Claimed prescription within 366 days before baseline date

*RAS* Renin-Angiotensin system

Unadjusted cox regression model for heart valve disease yielded a HR of 1.10 (95% CI 0.37–3.25, *p* = 0.87), while adjusted analysis yielded a HR of 0.71 (95% CI 0.21–2.4, *p* = 0.59), for patients claiming five or more bromocriptine prescriptions vs those claiming less than five prescriptions.

Patients that eventually were diagnosed with heart valve disease diagnosis (n = 16) claimed an accumulated median dosage of 275 mg (Q1-Q3: 150–1456 mg) bromocriptine and patients without claimed an accumulated median dosage of 200 mg (Q1-Q3: 150–525 mg) bromocriptine, *p* = 0.56.

## Discussion

In this nationwide cohort study of women who initiated treatment with bromocriptine, we observed a low incidence of heart valve disease during long-term follow-up, that did not differ significantly compared to matched controls from the background population. In a sensitivity analysis of patients who claimed five or more prescriptions of bromocriptine, the absolute risk of heart valve disease was somewhat higher, which could suggest a dose–response association, even if incidence did not differ significantly compared to matched controls. Still, the absolute risk of heart valve disease remained low, and this patient group was also characterized by a higher prevalence of conventional risk factors for cardiovascular disease. Furthermore, bromocriptine treated women who eventually were diagnosed with heart valve disease did not receive higher accumulated dosage of claimed bromocriptine compared to those who did not. This was also in line with a prior study, in which a more heterogenous patient population received much higher accumulated doses of bromocriptine (median 7815 mg over 49 months) without any signal of excess risk of heart valve disease.[20].

To date, most research have focused on the risk of heart valve disease associated with the dopamine agonist cabergoline and only few studies have investigated bromocriptine. Prior to this raised awareness, cabergoline was more widely used in the treatment of hyperprolactinemia due to its better tolerability, therapeutic efficacy and dosage advantages [9]. Previously, three smaller studies have not found any association between long-term bromocriptine-use and clinically significant heart valve disease [13, 14, 21]. Steffensen et al. investigated the incidence of heart valve abnormalities in Danish patients with hyperprolactinemia irrespective of treatment. They found low incidence and no significant differences between patients (0.80%) and controls (0.31%) after a median of 16 years of follow-up [22]. Thus, our knowledge regarding the association of bromocriptine use and heart valve disease in patients with hyperprolactinaemia is sparse and based on subgroups of few patients.

Another important finding of our study was a significantly higher mortality rate for patients with hyperprolactinemia than controls, not readily explained by heart valve disease incidence. The higher mortality observed could be related to the underlying disease, transsphenoidal surgery for pituitary adenomas, prolactin’s many diverse functions [23] or unmeasured confounders. The same tendency with a higher all-cause but also cardiovascular mortality in hyperprolactinaemic patients has been reported previously [24, 25]. In our sensitivity analysis with additional matching of comorbidity and pharmacotherapy, mortality remained increased in patients with hyperprolactinemia, although to a lesser degree than in the primary analysis (*p* = 0.02 vs. *p* = 0.002).

In our study, the 10-year incidence of heart valve disease and surgery was low in both bromocriptine treated patients and matched controls (Figs. 2a and 3a). Furthermore, previous studies found that heart valve regurgitation occurs not uncommonly in people with structurally normal hearts [26–29]. In 1968, World Health Organization (WHO) published 10 principles that should be considered when making screening guidelines [30, 31]. Taken together, we believe that the clinical yield of echocardiographic screening in bromocriptine-treated female patients is low, and our findings are in line with the recommendations of not screening these patients as stated in current European guidelines [12].

### Limitations

The main limitations of the present study are the absence of important clinical variables, such as body mass index, smoking, left ventricular ejection fraction, and systolic blood pressure. Another potential limitation was the definition of disease based on prescriptions rather than diagnostic coding. To accommodate this, we performed sensitivity analysis comparing baseline characteristics and risk of heart valve disease of bromocriptine treated patients with and without a diagnosis of hyperprolactinaemia. We only included women in the present study and it remains unknown whether our findings regarding safety of bromocriptine use extend to men. Residual confounding or confounding by indication could as well be a limitation in our study.

## Conclusions

Initiation of treatment with bromocriptine in younger and otherwise healthy women was not associated with an increased long-term risk of heart valve disease compared to age and sex-matched controls, although it was associated with an increased mortality which may at least partly be explained by higher burden of comorbidity. Our study suggests a low clinical yield of echocardiographic screening in bromocriptine treated women which is in accordance with current guidelines.

## Supplementary Information

**Additional file 1.** **Supplementary Appendix. Supplementary Table 1:** Baseline characteristics for bromocriptine treated patients and controls matched on age, sex, major comorbidity and pharmacotherapy. **Supplementary Appendix Table 2:** ICD-10 and -8 codes used to identify in and outpatient diagnoses, and procedure codes according to the Nordic Medico-Statistical Committee nomenclature. **Supplementary appendix Table 3:** ATC codes for evaluated pharmacotherapy.

## Data Availability

The datasets generated and/or analysed during the current study are not publicly available due to Danish national policy on availability of health care registers, but are available from the corresponding author on reasonable request.
