# Supplementary material for: Incidence of heart valve disease in women treated with the ergot-derived dopamine agonist bromocriptine
Source: BMC Cardiovasc Disord. 2021 Dec 28;21:622. doi: 10.1186/s12872-021-02439-y (PMC8714426; doi:10.1186/s12872-021-02439-y)
Supplement: Supplementary file 1 — Additional file 1. Supplementary Appendix. Supplementary Table 1: Baseline characteristics for bromocriptine treated patients and controls matched on age, sex, major comorbidity and pharmacotherapy. Supplementary Appendix Table 2: ICD-10 and -8 codes used to identify in and outpatient diagnoses, and procedure codes according to the Nordic Medico-Statistical Committee nomenclature. Supplementary appendix Table 3: ATC codes for evaluated pharmacotherapy. [file 12872_2021_2439_MOESM1_ESM.docx]

**Title:** Incidence of heart valve disease in women treated with the ergot-derived dopamine agonist bromocriptine

**Short title**: Bromocriptine and heart valve disease

**Authors:** Marianne F. Clausen MB^1^

Rasmus Rørth MD^1^

Christian Torp-Pedersen MD DMSc^2^

Lucas Malta Westergaard MB^1^

Peter E. Weeke MD PhD^1^

Gunnar Gislason MD PhD^3^

Lars Køber MD DMSc^1^

Emil Fosbøl MD PhD^1^

Søren Lund Kristensen MD PhD^1^

**Affiliations:** ^1^Department of Cardiology, Rigshospitalet, University of Copenhagen, Copenhagen, Denmark; ^2^Department of Clinical Investigation and Cardiology, Nordsjaellands Hospital, Hilleroed, Denmark; ^3^Department of Cardiology, Gentofte/Herlev University Hospital, Copenhagen, Denmark;

**Correspondence:** Søren Lund Kristensen, MD PhD

Department of Cardiology, Rigshospitalet

Blegdamsvej 9, Copenhagen 2100, Denmark

Tel: +45 28694385

E-mail: slk@heart.dk

**Keywords:** Bromocriptine, ergot-derived dopamine agonist, heart valve disease, hyperprolactinemia

## Supplementary Appendix

Supplementary Table 1: Baseline characteristics for bromocriptine treated patients and controls matched on age, sex, major comorbidity and pharmacotherapy


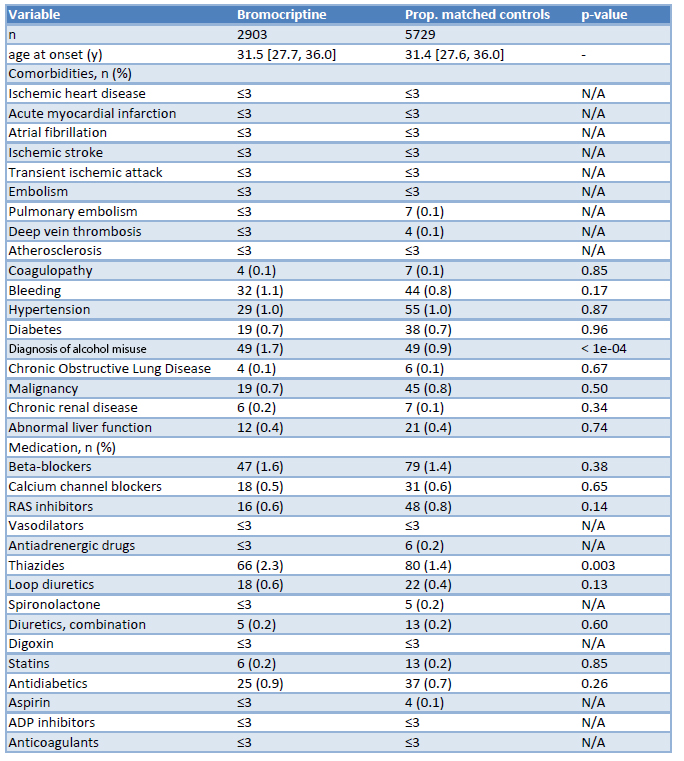


Patients were matched on age, sex, prior diabetes, deep venous thrombosis, bleeding, chronic renal failure, ischemic heart disease, stroke, COLD and ongoing use of beta blockers, loop diuretics and aspirin.

Due to the Danish Act on Processing of Personal Data, we cannot report any number less than four observations.

P-values were obtained by chi-square test for comparison. IQR = Interquartile range

Pharmaceuticals: Claimed prescription within 366 days before baseline date. RAS: Renin-Angiotensin system

Supplementary Appendix Table 2: ICD-10 and -8 codes used to identify in and outpatient diagnoses, and procedure codes according to the Nordic Medico-Statistical Committee nomenclature:

| The Danish National Patient Register:  Inclusion diagnoses:  Hyperprolactinaemic disorders (BKHH, DN645A, E220, N62, E282, N644, E221, D352, D443)  Exclusion diagnoses:  Rheumatic heart disease (DI05-DI09 (390-398))  Rheumatic fever (DI00-DI02)  Cardiomyopathies or Chorda tendinae rupture (DI42, DI43, DI511 (425))  Non-rheumatic heart valve diseases (DI34-DI37 (424))  Congenital heart diseases (DQ23, DQ24 (74660-74669))  Endocarditis (DI33, DI38, DI39 (421))  Parkinson’s disease (DG20-DG22 (34299, 06600))  Heart failure (DI42, DI50, DJ819, DI110, DI130, DI132 (425, 428, 4270, 4271))  Outcomes:  Heart valve disease - diagnosis (DI34, DI35, DI36, DI37)  Valvular heart surgery - procedure (KFGA, KFGC-E, KFGW, KFJE, KFJF, KFK, KFM)    Comorbidities:  Ischemic heart disease (DI20-DI25 (410-414))  Acute myocardial infarction (DI21-DI22 (410))  Atrial fibrillation (DI48 (42793, 42794))  Ischemic stroke (DI63-DI64 (430-434, 436))  Transient Ischemic Attack (DG45 (435))  Embolism (DI74 (444))  Pulmonary embolism (DI26 (450))  Deep vein thrombosis (DI801-DI803, DI808, DI809, DI821-DI823, DI828, DI829 (45100, 45108, 45109, 45190, 45199, 45300, 45302-45304, 45309))  Atherosclerosis (DI70 (440))  Coagulopathy (DD66-DD69 (286))  Bleeding (DI60-DI62, DN02, DR31, DR04, DD62, DH052A, DG951A, DS368D, DK298A, DK228F, DI864A, DK638B, DK638C, DK638F, DK868G, DI312, DH356, DH431, DH450, DS064-DS066, DJ942, DD500, DK250, DK252, DK254, DK256, DK260, DK262, DK264, DK266, DK270, DK272, DK274, DK276, DK280, DK282, DK284, DK286, DK290, DK661, DK921, DK922, DI850)  Diabetes (DE10-DE14 (250))  Alcohol (DF10, DK70, DE52, DT51, DK860, DE244, DG312, DI426, DO354, DZ714, DG621, DG721, DK292, DL278A (291, 303, 57109, 57110, 57710))  Chronic Obstructive Lung Disease (DJ42-DJ44 (490-492))  Malignancy (DC00-DC97 (140-209))  Abnormal liver function (DB15-DB19, DK70-DK77, DC22, DI982, DZ944, DD684C, DQ618A (571-573, 155, 070))  Chronic renal disease (DN02-DN08, DN11-DN12, DN14, DN18-DN19, DN26, DM321B, DN158-DN160, DN162, DN153, DN164, DN168, DQ612, DQ613, DQ615, DQ619, DE102, DE112, DE132, DE142, DI120, DM300, DM313, DM319, DT858, DT859, DZ992 (403, 404, 581-584)) |
| --- |

Supplementary appendix Table 3: ATC codes for evaluated pharmacotherapy

Inclusion criteria

Bromocriptine (N04BC01)

Exclusion criteria

Fenfluramine (A08AA02)

Dexfenfluramine (A08AA04)

Ergotamine (N02CA02)

Levodopa (N04BA01-06)

Cabergoline (G02CB03)

Quinagolide (G02CB04)

Pergolide (N04BC02)

Baseline medication:

Beta-blockers (C07, C09BX)

Calcium channel blockers (C08, C07F, C09BB, C09DB)

RAS inhibitors (C09)

Vasodilators (C02DB, C02DD, C02DG)

Antiadrenergic drugs (C02A, C02B, C02C)

Thiazides (C03A, C07B, C07D, C09XA52, C03EA01)

Loop diuretics (C03C, C03EB01, C03EB02)

Spironolactone (C03DA01)

Diuretics, combination (C07C, C08G, C03B, C09BA, C09DA)

Digoxin (C01AA05)

Statins (C10AA)

Antidiabetics (A10)

Aspirin (B01AC06)

ADP inhibitors (B01AC04, B01AC22, B01AC24)

Anticoagulants (B01AA, B01AE, B01AF)

Hypertension: Defined as the use of 2 or more of following drug agents: beta blockers, calcium channel blockers, RAS inhibitors, vasodilators, antiadrenergic drugs, thiazides, loop diuretics, spironolactone, diuretics in combination
